# Brainstem Evoked Auditory Potentials with speech stimulus in the Auditory Processing Disorder

**DOI:** 10.1016/S1808-8694(15)30665-0

**Published:** 2015-10-19

**Authors:** Renata Filippini, Eliane Schochat

**Affiliations:** 1 Speech and hearing therapist, University of São Paulo Medical School, Human Communications - Rehabilitation Sciences program; 2 PhD; Associate Professor - Speech and Hearing Therapy Program - Universit of São Paulo. Department of Speech and Hearing Therapy, Occupational Therapy and Physical Therapy - University of São Paulo Medical School - FMUSP

**Keywords:** speech perception, auditory evoked potentials, auditory perceptual disorders

## Abstract

Although the clinical use of click stimuli to assess auditory function at the brainstem is already established, and numerous research projects use such stimuli to study human hearing, little is known about the auditory processing of a complex stimulus like speech.

**Aim:**

This study aimed at validating the speech stimulus as an effective method to evaluate speech auditory processing, to help us better understand its disorders.

**Materials and Methods:**

This prospective clinical study tested 20 subjects with Auditory Processing Disorders (APD) and 20 subjects with normal development (ND - control group) using the Brainstem Auditory Evoked Potentials with clicks and speech stimuli. The latter is based on first 40ms of the spoken syllable /da/.

**Results:**

No differences were observed between the groups regarding the click stimulus. However, with the speech stimulus the APD group presented latency delay and lower amplitudes when compared to the ND group.

**Conclusion:**

Speech stimulus proved to be more sensitive for the evaluation of Auditory Processing Disorders, showing possible alterations in synchronicity and speech processing neural input speed, especially as to the linguistic information of the latter.

## INTRODUCTION

The auditory system integrity, from the sound signal capture at the outer ear all the way to its cortex interpretation, reflect on the normal development of language, speech, reading and writing^1^,^2^. The detailed knowledge about the underlying process to “hearing and understanding” must provide relevant data from the understanding of disorders associated with those areas, as well as for the choice of adequate approaches.

We know today that children with difficulties to follow oral instructions and to understand fast and degraded speech may have some hearing loss; however, a significant number of them will have hearing thresholds within normal ranges, and their hearing problems will be the result of a hearing deficit in perception. Such children with difficulties to process hearing information despite the integrity of the system are believed to have an auditory processing disorder (APD) ^3^.

The term auditory processing (AP) refers to auditory processing information in the central nervous system (CNS) and to the neurobiological activity underlying this processing^4^.

According to Kraus & Nicol^5^ in auditory processing disorders the speech auditory perception may be impaired because it is a complex acoustic signal which demands much from the auditory system, which must be sensitive to quick spectrum changes, to the unfavorable signal/noise ratio and to the reception of many stimuli in a short time span. The authors also stated that the processing required for speech perception has a substantially automatic base and does not depend on higher cognitive elements, in other words, it would mostly in the brainstem (BS). A lesion in this region of the auditory pathways could then be responsible for many difficulties associated with understanding speech.

The assessment of auditory pathway integrity in the BS is done by means of the Brainstem Evoked Auditory Potential (BEAP). BEAP records the bioelectrical activities associated with auditory stimuli. The acoustic stimulus most often employed to obtain BEAP is the click, since it triggers a synchronic response from a large number of neurons and has a broad frequency^6^. However, Russo et al.^7^ consider stimuli like the click and pure tone, although broadly used in clinical practice, as simple stimuli; and responses to complex stimuli, such as speech, are much less understood.

Much research is being carried out aiming at outlining speech auditory processing, reporting on the speech stimuli response in the BS^7^, ^8^, ^9^, ^10^, ^11^, ^12^, ^13^, ^14^, ^15^, as well as its relationship with cerebral cortex processing^8^,^13^,^16^,^17^, the efficacy of auditory training in the rehabilitation of patients with speech perception deficits^10^,^18^ beyond the effect masking has on answers^9^,^7^.

Russo et al.^7^ suggest that brainstem responses generate direct information on the sound structure of a spoken syllable, and it is decoded by the auditory system. Johnson et al.^12^ believe that the BS responses for speech stimuli is a method that can be used to assess subcortical auditory processing and can be used as a biological marker of the deficient sound decoding.

**Chart 1:**
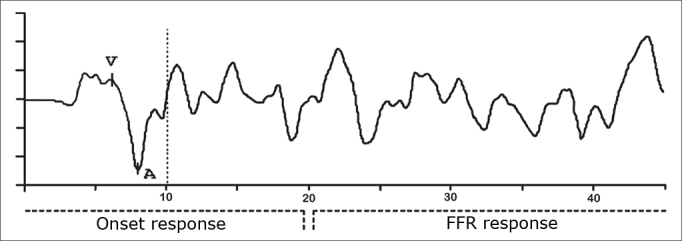
Representation of the Brainstem response for speech stimulus in an individual from the DT group in this study.

It is important to consider that the specific aspects of the acoustic signal structure are maintained and reflected in neural coding. Thus, similar to the syllable, the BS response to speech (Chart 1) can be divided in a transient portion and a sustained portion, respectively - the onset response component and FFR (frequency-following response).

Onset responses represent, primarily the response to the stimulus onset and to the successive modulations caused by vocal fold vibrations, and the FFR reflects the harmonic structure of the vowel which remains during the reproduction of a periodic stimulus and shows the general integrity of the response in relation to itself^7^,^14^.

The speech stimulus generates, necessarily before 10ms, a complex trace that includes a positive peak (V wave analogue to the V wave produced by the click) immediately followed by a negative peak (wave A). Among the many negative peaks that appear after the VA complex, the most frequent and stable ones are the C and F peaks. These waves are analyzed as to their absolute amplitude and latencies and the analysis of the latency, amplitude and slope (VA amplitude/VA duration) of the VA complex^12^.

Another way of analyzing the BS response to speech, in qualitative terms, considers the Acoustic Theory of Speech Production, by Fant^19^. According to this theory, speech waves are a response from a filter system to one or more sound sources, and it is possible to discuss the sound waves specifically in terms of source characteristics (larynx) and filters (vocal tract). According to Johnson et al.^12^, the source characteristics generate para-linguistic information and the filtering system generates the speech linguistic information, and these would be represented respectively by the FFR and onset responses.

Wible et al.^17^ and Abrams et al.^13^ found an intimate relation between the brainstem and cortical potentials, suggesting that the greater synchronicity of the transient acoustic information coding in the brainstem contributes to a more robust processing at cortical levels. In other words, the latency deficits in BS responses for speech stimuli have a negative impact on the processing of fast acoustic signals by cortex specialized structures.

This study aimed at testing the validity of the speech stimulus in the assessment of the auditory function in the BS by means of the BEAP, in individuals with normal development as well as in individuals with auditory processing disorder, starting research projects in Brazil to try and outline the speech auditory processing allowing for a better understanding of the disorders associated with it.

## MATERIALS AND METHODS

The present study was approved by the Ethics Committee under protocol # 527/04.

### Series

40 individuals between 7 and 24 years of age were part of the study. Of these, 20 complained of difficulties associated with auditory processing, and had alterations in their assessments, making up the study group (TPA). The remaining (20) individuals were invited to participate in the study because they did not have complaints associated with auditory processing, learning or language, and did not show alterations in the auditory processing assessment, making up the control group (DT).

### Stimuli and procedures

The subjects from both groups were tested as to BEAP responses for clicks and speech stimulus. The responses were recorded by means of the Traveler Express portable system by BioLogic.

We inspected the external acoustic canal using the otoscope in order to check for the presence of any factor that could prevent the exam from being performed. After skin cleaning with abrasive paste, the electrodes were placed on the vertex and on the right and left mastoids, using electrolytic paste and adhesive tape. Impedance values for the electrodes were below 5 KWs.

The click stimulus was presented to the right ear by mean of earphones at a rate of 19 stimuli per second, and we had 2,000 stimuli at 80 dBnHL being produced. The recording window was of 10 milliseconds and the stimuli were filtered from 100 to 3,000 Hz. A second stimulation was carried out in order to produce and confirm the wave trace. In this we identified and analyzed waves I, III and V.

For BEAP speech stimulation we had to produce such stimulus. We chose a natural stimulus instead of the synthesized one, thus the /da/ syllable was narrated in a recording studio by a male voice, recorded using the Sound Forge 6.0 (Sony) software and edited by the Vegas 4.0 (Sony) software, in such a way as to produce a stimulus with similar patterns to those described by King et al.^8^ and Wible et al.^11^. From the original syllable, only the five first formants were separated, which resulted in the 40 ms stimulus, with the transient portion of the syllable. Just like the stimulus used by Wible et al.^11^ - the vowel /a/ was shortened in order to increase the stimulation rate and, therefore, better activate the system.

The stimuli were organized in groups of 4, separated among themselves by 12 ms and, among each group of stimulus, the interval was of 30 ms. According to Wible et al.^11^ ^12^ ms is the shortest interstimuli interval that can be used without presenting a stimulus while the previous one is still active.

The speech stimulus was presented by means of headphones coupled to a disc-man, on the right ear at 80 dBH at a rate of 18 stimuli per second. A total of 3,000 stimuli were produced, 3 scans of 1,000 stimuli. The recording window was of 45 milliseconds.

The curves obtained in each scan were added up and on the resulting trace, waves V, A, C and F were identified and analyzed.

### Result Analysis

Analysis of waves I, III and V generated by the click stimulus was based on the conventional clinical analysis of the absolute latencies, interpeak latencies and amplitudes.

The analysis of waves V, A, C and F (chart 1) generated by the speech stimulus was based on the descriptions from Johnson et al.^12^.

The values for both stimuli went through statistical analysis in which the mean, median, standard deviation, minimum, maximum and confidence interval values were calculated for the sample. In order to check whether or not there were statistically significant differences regarding the results found in both groups, we studied the p value. The p value was calculated by means of the T student test and Variance Analysis (ANOVA) with significance levels of 5% (P < 0.05).

## RESULTS

### Click

The trace analysis of all the subjects for click stimulus showed the presence of waves I, III and V with latency values as well as interpeak values within normal standards, according to Hall's parameters^20^. These data showed auditory pathway intactness all the way to the BS in all the subjects (n = 40).

When we compared the values of latency, amplitude and interpeak intervals (Table 1) we did not observe significant differences (p Value > 0.05) between the DT and TPA groups, and the averages of waves I, III and V latency values were very close. We noticed a trend towards having significant differences between the groups as to the mean values of the Interpeak I-III interval.

**Table 1:** Click stimulus - minimum, maximum, mean, medians, standard deviation (SD), confidence interval and latency p values of the latencies, amplitudes and interpeak intervals in both groups.

CLICK		N	Values		Mean	Median	SD	Thresholds		P value	
			Min	Max				Min	Max	T test	ANOVA
	DPA	20	1,12	1,72	1,48	1,48	0,14	1,41	1,55		
LAT I										0,8637	0,7051
	DT	20	1,24	1,84	1,49	1,45	0,15	1,42	1,56		
	DPA	20	3,16	4,04	3,60	3,57	0,23	3,49	3,71		
LAT III										0,2134	0,2130
	DT	20	3,28	3,88	3,52	3,46	0,18	3,44	3,60		
	DPA	20	5,12	5,8	5,44	5,46	0,19	5,35	5,53		
LAT V										0,6063	0,6063
	DT	20	5,12	5,68	5,41	5,39	0,16	5,33	5,49		
	DPA	20	1,84	2,48	2,13	2,08	0,18	2,05	2,21		
LAT I-III										0,0615	0,0615
	DT	20	1,8	2,24	2,04	2,04	0,11	1,99	2,09		
LAT III-V	DPA	20	1,36	2,04	1,83	1,88	0,16	1,75	1,91	0,2006	0,2000
	DT	20	1,72	2,12	1,89	1,86	0,12	1,83	1,95		
LAT I-V	DPA	20	3,6	4,32	3,97	3,96	0,17	3,89	4,05	0,5012	0,5000
	DT	20	3,72	4,12	3,94	3,92	0,09	3,90	3,98		
AMP I	DPA	20	-0,71	0,51	0,16	0,21	0,27	0,04	0,28	0,8047	0,8040
	DT	20	-0,1	0,4	0,17	0,17	0,10	0,12	0,22		
AMP III	DPA	20	-0,48	0,63	0,30	0,33	0,25	0,18	0,42	0,1875	0,1834
	DT	20	0,19	0,6	0,38	0,38	0,10	0,33	0,43		
AMP V	DPA	20	-0,59	0,5	0,22	0,28	0,25	0,10	0,34	0,6322	0,6306
	DT	20	0,11	0,42	0,24	0,22	0,09	0,20	0,28		

Statistically significant differences (P value < 0.05)

### Speech Stimuli

For all the subjects, waves V, A, C and F were identified in the tracing resulting from the summation of the three scans performed. In one individual only (2.5%), belonging to the TPA group, it was not possible to identify wave F, because he did not have it or it was in a greater latency than that of the interval studied (45 ms). For this reason we analyzed only the responses from 19 individuals from the study group. Thus, the mean, standard deviation and p values of the latencies and amplitudes were calculated and analyzed (Table 2).

**Table 2:** Speech stimulus - minimum, maximum, mean, median, standard deviation (SD), confidence interval and p values of the latencies and amplitudes in both groups.

SPEECH		N	Values		Mean	Median	SD	Thresholds		P value	
			Min	Max				Min	Max	T test	ANOVA
LAT V	DPA	20	4,22	12,67	7,55	7,31	1,76	6,72	8,37	0,0331*	0,0314*
	DT	20	4,40	8,27	6,54	6,51	1,00	6,07	7,00		
LAT A	DPA	20	5,46	15,49	9,10	8,75	2,08	8,12	10,07	0,0452*	0,0428*
	DT	20	6,34	10,03	8,00	7,92	1,06	7,50	8,49		
LAT C	DPA	20	14,26	23,41	19,45	20,06	2,46	18,29	20,59	0,0719	0,0717
	DT	20	16,19	23,94	18,12	17,60	2,05	17,16	19,08		
LAT F	DPA	19	36,46	44,46	40,93	41,18	1,93	40,00	41,86	0,2721	0,2263
	DT	20	38,18	44,18	40,27	40,22	1,43	39,60	40,93		
AMP V	DPA	20	0,06	0,70	0,30	0,27	0,21	0,20	0,39	0,9359	0,9359
	DT	20	0,02	0,77	0,31	0,32	0,18	0,21	0,39		
AMP A	DPA	20	-0,80	-0,09	-0,43	-0,43	0,19	0,51	-0,33	0,0062*	0,0061*
	DT	20	-1,30	-0,30	-0,62	-0,58	0,23	0,72	-0,51		
AMP C	DPA	20	-0,75	-0,01	-0,31	-0,29	0,18	0,39	-0,22	0,0043*	0,0042*
	DT	20	-0,89	-0,16	-0,50	-0,51	0,22	0,60	-0,39		
AMP F	DPA	19	-0,46	-0,04	-0,24	-0,20	0,13	0,30	-0,17	0,0592	0,0606
	DT	20	-1,06	0,01	-0,37	-0,31	0,26	0,48	-0,24		

* Statistically significant differences (P value < 0.05)

There was a significant difference (p Value < 0.05) between the DT and TPA groups for the latency values of waves V and A. For the C wave latency, we noticed only one trend towards significant difference between the groups. Now, as far as amplitudes are concerned, we observed significant differences between the DT and TPA groups for the values of waves A, C and F.

The analysis of the VA Complex (Table 3) in relation to the mean values of latency, amplitude and slope (VA amplitude VA/ VA duration) showed a significant difference between the DT and TPA groups in regards of the VA complex amplitude values and, consequently, of slope.

**Table 3:** Speech stimulus: VA Complex - minimum, maximum, mean, median, standard deviation (SD), confidence interval and p values for latency, amplitude and slope in both groups.

AV Complex		N	Values		Mean	Median	SD	Thresholds		P value	
			Min	Max				Min	Max	T test	ANOVA
LAT	DPA	20	0,71	3,17	1,55	1,40	0,65	1,25	1,85	0,6474	0,6472
	DT	20	0,88	2,64	1,46	1,28	0,51	1,22	1,70		
AMP	DPA	20	0,25	1,44	0,72	0,68	0,29	0,58	0,86	0,0246*	0,0246*
	DT	20	0,50	1,38	0,93	0,96	0,26	0,80	1,06		
SLOPE	DPA	20	0,22	1,36	0,51	0,38	0,25	0,39	0,63	0,0147*	0,0147*
	DT	20	0,28	1,18	0,71	0,43	0,26	0,59	0,83		

* Statistically significant differences (P value < 0.05)

## DISCUSSION

In relation to the individuals under study, we must stress that the large age range of the individuals participating in the study was not a problem, since the expectation is that the auditory pathway at the level of the brainstem of a two-year old child already presents responses just like those from adults^20^.

### Click

In this study we observed normal response values for clicks, both for the DT and TPA group, without significant differences between the groups, leading us to believe that individuals with TPA process sounds such as the click, at the level of the BS, in a similar fashion to that of individuals with normal development.

Hall and Johnson^21^ state that, for individuals with TPA, the proportion of abnormalities in BEAP is much lower than that in the middle and long latency potentials, which strengthens our previous assumption.

Song et al.^14^ investigated the responses for clicks between a group of children with normal learning and a group of children with learning disorder. No significant differences were found between the groups, not even when contralateral masking was introduced. In this case, the amplitudes of both groups were similarly reduced.

Thus, we can state that individuals with auditory processing disorders did not have lesions in their auditory pathway all the way to the brainstem or, even, did not have difficulties in processing simple acoustic information such as the click.

### TPA Group - Speech Stimulus

In this study, for the TPA group in relation to the DT, speech stimuli responses with latency of waves A, C and the VA complex were significantly reduced, and also there was a lower VA complex slope. We can then suggest that children with TPA may have changes in the response generator synchronization (amplitude differences) and/or in the nervous signaling transmission velocity (latency differences)^11^. Other researchers^5^,^8^,^11^ studied the BEAPs for speech stimuli in subjects with normal development and those with learning difficulties and found results similar to the ones we did. King et al.^8^ noticed significant differences in relation to the A wave latency and suggested that at least some of the children with learning disorders have abnormalities in the acoustic representation of speech sounds in the low brainstem.

Kraus & Nicol^5^ found significantly increased onset response latencies (waves V, A and C) in children with learning disorders when compared to their counterparts with normal development. Wible et al.^11^ observed that the children with learning disorders had lower VA complex slope when compared to children with normal development.

When commenting the workshop “Speech Perception Nature “, Kraus & Nicol^5^ mention that: speech perception involves peripheral auditory analyses, automatic extraction of the brainstem nuclei characteristics, which lead both to the classification of words and phonemes. Thus, the alterations observed in speech processing at the BS could represent more specific alterations at a cortical level.

Although this relationship was not observed in this study, because cortical evaluations were not carried out, Wible et al.^17^ concluded that a greater coding synchronicity of the speech transient aspects (onset responses, waves V, A and C) at the level of BS contribute to a more robust processing at a cortical level, and vice-versa. Therefore, a BS alteration can suggest difficulties in speech cortical processing and, consequently, in the very development of speech and language.

This relationship becomes even clearer with the analysis of response alterations for speech in the BS based on Fant's Acoustic Theory of Speech Production^19^, as suggested by Kraus & Nicol^22^ and Johnson et al.^12^. Since the alterations noticed happened to waves V, A and C, which reflected the system's response to the speech filtering capacity, the difficulties found by TPA children for speech processing would happen in relation to linguistic aspects.

Johnson et al.^12^ suggest that the BS measures associated to the information generated by the speech filter may function as biological markers of neural asynchronicity in children with learning disorders such as dyslexia or in children with auditory processing disorder.

Therefore, based on the aforementioned, it would not be too difficult to state that the speech processing alteration in the BS causes a failed development in linguistic skills.

### Speech Stimulus -DT Group

Although the study's goal was not to define normality parameters, we compared the DT group responses for speech stimulus with the parameters obtained by Russo et al.^7^ (Chart 2). The values found are similar, without significant differences, although in our study they are slightly increased, which can be justified by mild differences in response collection and analysis.

**Chart 2:** Comparing mean latency and amplitude values found in the present study and in the study by Russo et al.*.

	PRESENT STUDY	RUSSO 2004*
LAT V	6,53	6,61
LAT A	8	7,51
LAT VA	1,46	0,89
LAT C	18,12	17,69
LAT F	40,26	39,73
AMP V	0,3	0,31
AMP A	-0,62	-0,65
AMP VA	0,93	0,97
AMP C	-0,49	-0,36
AMP F	-0,36	-0,43

* Russo N, Nicol T, Musacchia G, Kraus N. Brainstem Responses to Speech Syllable. Clinical Neurophysiology 2004; 115: 2021 – 30.

The results obtained from this study showed in the TPA group possible alterations as to neural signaling velocity and synchronicity in the processing of complex stimuli like speech, especially as to linguistic information.

Further studies must be carried out comparing the relationship of speech responses at the brainstem with those at a cortical level, and also the relationship between the electrophysiological responses and the behavioral ones, seeking a better understanding of the auditory processing of complex signals like speech.

Another goal that must be pursued associated with responses to complex stimuli at the brainstem level is the relationship with language disorders, speech, learning and auditory processing and the consequences of auditory treatment and Speech and Hearing Therapy in speech processing.

## CONCLUSION

Since we did not obtain clear and significant differences among the response values for clicks in groups TPA and DT, and we did not notice relations between this one and the speech stimulus, leading us to conclude that the click stimulus does not bring precise information on the auditory processing of complex acoustic signals at the brainstem, and that the speech stimulus was more sensitive for this type of approach because significant differences were found among the responses from groups TPA and DT for this stimulus. Thus, the Brainstem Evoked Auditory Potential with a speech stimulus proved to be a valid method in the assessment of Brainstem auditory function in individuals with auditory processing disorder.
